# Recombinant Noroviruses Circulating in Spain from 2016 to 2020 and Proposal of Two Novel Genotypes within Genogroup I

**DOI:** 10.1128/spectrum.02505-21

**Published:** 2022-07-13

**Authors:** Noemi Navarro-Lleó, Cristina Santiso-Bellón, Susana Vila-Vicent, Noelia Carmona-Vicente, Roberto Gozalbo-Rovira, Roberto Cárcamo-Calvo, Jesús Rodríguez-Díaz, Javier Buesa

**Affiliations:** a Department of Microbiology, School of Medicine and Dentistry, University of Valencia, Valencia, Spain; b INCLIVA, Hospital Clínico Universitario de Valencia, Valencia, Spain; U.S. Food and Drug Administration

**Keywords:** genogroups, genotypes, molecular surveillance, noroviruses, phylogenetic analysis, recombinant strains

## Abstract

Noroviruses are the leading cause of sporadic cases and outbreaks of viral gastroenteritis. For more than 20 years, most norovirus infections have been caused by the pandemic genotype GII.4, yet recent studies have reported the emergence of recombinant strains in many countries. In the present study, 4,950 stool samples collected between January 2016 and April 2020 in Valencia, Spain, from patients with acute gastroenteritis were analyzed to investigate the etiological agent. Norovirus was the most frequently detected enteric virus, with a positivity rate of 9.5% (471/4,950). Among 224 norovirus strains characterized, 175 belonged to genogroup II (GII) and 49 belonged to GI. Using dual genotyping based on sequencing of the open reading frame 1 (ORF1)/ORF2 junction region, we detected 25 different capsid-polymerase-type associations. The most common GII capsid genotype was GII.4 Sydney 2012, followed by GII.2, GII.3, GII.6, and GII.17. A high prevalence of recombinant strains (90.4%) was observed among GII infections between 2018 and 2020. GII.4 Sydney[P16] was the predominant genotype from 2019 to 2020. In addition, GII.P16 polymerase was found harbored within six different capsid genes. GI.4 and GI.3 were the predominant genotypes in genogroup I, in which recombinant strains were also found, such as GI.3[P10], GI.3[P13], and GI.5[P4]. Interestingly, applying the criterion of 2 times the standard deviation, we found that 12 sequences initially classified as GI.3 may represent two new tentative genotypes in genogroup I, designated GI.10 and GI.11. This study shows the extensive diversity of recombinant noroviruses circulating in Spain and highlights the role of recombination events in the spread of noroviruses.

**IMPORTANCE** Human noroviruses are the most common cause of viral diarrhea. There are no approved vaccines to prevent their infections yet, which would be very useful to protect infants, small children, and the elderly in residential institutions. These viruses are extremely contagious and can be transmitted by contaminated food and water as well as directly from person to person. Molecular surveillance and epidemiology of norovirus infections allow the identification of the most common viral strains in different geographical areas over time. Noroviruses show wide genetic variability due to a high rate of mutations but also due to genomic recombinations, as we demonstrate in this study. We have detected 25 different viral capsid-polymerase gene associations among 224 norovirus strains characterized in Spain between January 2016 and April 2020, including two tentative new capsid genotypes in genogroup I.

## INTRODUCTION

Norovirus (NoV) infections are considered a primary cause of acute gastroenteritis (AGE) in all age groups worldwide (1, 2) and lead to a major health problem. In developed countries, norovirus outbreaks bring a high economic burden, estimated at over $64 billion annually (3). The settings most affected are hospitals (4), restaurants (5), cruise ships (6), schools (7), and nursing homes (8). In countries where rotavirus vaccination has been implemented, noroviruses have become a major cause of gastroenteritis in children (9–11). These viruses are highly contagious and are transmitted from person to person and by contaminated food or water (12). It has been estimated that noroviruses cause between 70,000 and 200,000 deaths annually in developing countries (2, 13).

The genus *Norovirus* belongs to the family *Caliciviridae*, members of which are nonenveloped viruses with a single-stranded positive-sense RNA genome of approximately 7.5 kb in length, organized into three open reading frames (ORFs). ORF1 encodes a polyprotein that is posttranslationally cleaved into six nonstructural viral proteins, including the RNA-dependent RNA polymerase (RdRp). ORF2 and ORF3 encode the major (VP1) and minor (VP2) capsid proteins, respectively (14). VP1 is organized into the shell (S) and protruding (P) domains (13). The P domain can be further divided into the P1 and P2 subdomains. The P1 subdomain forms the anchoring portion of the P dimer, connecting it to the S domain, while the highly variable P2 subdomain is the most surface-exposed region of the norovirus capsid. The P2 subdomain acts as the target for neutralizing antibodies and contains carbohydrate binding sites that enable human norovirus infections (15, 16).

Based on phylogenetic analyses of the complete VP1 amino acid sequences, noroviruses have been classified into 10 distinct genogroups (genogroup I [GI] to GX), which are subdivided into 49 different genotypes (17). Of these, GI, GII, and GIV strains cause illness in humans, and the majority of diseases are correlated with GI and GII infections.

Norovirus diversity arises through evolutionary mechanisms such as recombination or the accumulation of mutations (18). Recombination often occurs at the ORF1/ORF2 junction, leading to new combinations of capsid and polymerase genotypes, which contribute to increasing genetic diversity (18–20). As a result, these new recombinant strains might have increased fitness, pathogenicity, and/or transmissibility compared to their ancestor strains (21–23). Furthermore, the same capsid harbors different polymerase genotypes, which may offer an advantage by changing the efficiency of virus replication (24). Despite the high genetic diversity of noroviruses, during recent decades, a single genotype (GII.4) has been the most prevalent in humans worldwide (25). This virus is highly versatile, and new variants emerge rapidly due to antigenic drift in VP1 and genetic recombination between circulating norovirus strains (20, 26).

To understand the epidemiology and genotypic trends of evolving noroviruses, we studied and characterized the molecular epidemiology of norovirus strains causing sporadic cases or outbreaks of AGE in the Valencian Community, Spain, from January 2016 to April 2020.

## RESULTS

### Norovirus detection and typing.

In total, 9.5% (471/4,950) of stool specimens tested norovirus positive by conventional reverse transcription (RT)-PCR and/or RT-quantitative PCR (qPCR). Of these, 264 viral strains could be further amplified for sequencing, genogrouping, and genotyping, which we eventually achieved for 224 strains (84.8%). Their polymerase and/or capsid genotypes were determined: 49 (21.9%) strains belonged to genogroup I (GI), and 175 (78.1%) strains belonged to GII.

### Genetic diversity of noroviruses.

A wide range of norovirus genotypes was detected throughout the 5-year study period (Table 1). Six capsid genotypes were identified within GI (GI.1 and GI.3 to GI.7), and 14 capsid genotypes were identified in GII (GII.1 to GII.7, GII.10, GII.12 to GII.14, GII.17, GII.20, and GII.21). The most prevalent GI genotype was GI.4 (13/40; 32.5%), which was involved in four different outbreaks occurring in March 2018 and 2019. Strains showing the GI.3 capsid gene represented the second most frequent genotype (10/40; 25%), and it was found mainly from 2018 onward. In genogroup II, GII.4 viruses caused 47.9% (58/121) of AGE cases. The GII.4 Sydney variant represented 50.8% (31/61) of norovirus strains detected in 2019. There were 7 GII.4 isolates that initially could not be assigned to a specific variant, which were associated with P31 polymerases. Other frequently identified genotypes in this study were GII.2, GII.6, and GII.17 at the same ratio (12/121; 9.9%) and GII.3 (7/121; 5.8%). It is noteworthy that GII.17 was detected late in 2018. The temporal distribution of polymerase genotypes showed that GII.P17 and GII.P4 predominated from 2016 to 2018 with the same prevalence (19/84; 22.6%) and were replaced by GII.P16 (42/72; 58.3%) from 2019 onward (Table 1). Furthermore, GII.P4 New Orleans and GII.P31 were widely detected in the same proportion (12.2%) throughout the study period.

**TABLE 1:** Norovirus genotypes producing sporadic cases or outbreaks of gastroenteritis in the Valencian Community, Spain, from January 2016 to April 2020^*a*^

Genotype	No. (%) of isolates					
	2016	2017	2018	2019	2020	Total
ORF1/ORF2						
GI.1[P1]	2 (2.4)			1 (1.4)	2 (10.0)	5 (2.2)
GI.3[P3]				2 (2.9)	4 (20.0)	6 (2.7)
GI.3[P10]			1 (2.9)			1 (0.4)
GI.3[P13]				1 (1.4)	2 (10.0)	3 (1.3)
GI.4[P4]			3 (8.8)	2 (2.9)		5 (2.2)
GI.5[P4]			1 (2.9)			1 (0.4)
GI.6[P11]			1 (2.9)			1 (0.4)
GI.7[P7]		1 (5.3)			2 (10.0)	3 (1.3)
GII.1[P33]		2 (10.5)				2 (0.9)
GII.2[P16]		1 (5.3)	1 (2.9)	5 (7.2)		7 (3.1)
GII.3[P12]				1 (1.4)		1 (0.4)
GII.3[P16]		1 (5.3)		1 (1.4)		2 (0.9)
GII.3[P21]			1 (2.9)			1 (0.4)
GII.3[P30]				3 (4.3)		3 (1.3)
GII.4[P4 New Orleans]	2 (2.4)	3 (15.8)		4 (5.8)	1 (5.0)	10 (4.5)
GII.4 Sydney[P4 New Orleans]	1 (1.2)					1 (0.4)
GII.4 Sydney[P16]				28 (40.6)		28 (12.5)
GII.4 Sydney[P31]	5 (6.1)			3 (4.3)		8 (3.6)
GII.4 unassigned[P31]	1 (1.2)			5 (7.2)	1 (5.0)	7 (3.1)
GII.5[P40]				1 (1.4)		1 (0.4)
GII.6[P7]	1 (1.2)			5 (7.2)	2 (10.0)	8 (3.6)
GII.7[P7]	1 (1.2)				1 (5.0)	2 (0.9)
GII.10[P16]				1 (1.4)	1 (5.0)	2 (0.9)
GII.12[P16]				1 (1.4)		1 (0.4)
GII.13[P16]				1 (1.4)	4 (20.0)	5 (2.2)
GII.13[P21]	1 (1.2)	2 (10.5)				3 (1.3)
GII.14[P7]				1 (1.4)		1 (0.4)
GII.17[P17]	3 (3.7)	1 (5.3)	1 (2.9)			5 (2.2)
GII.20[P20]				1 (1.4)		1 (0.4)
ORF1^*b*^						
GI.P1			1 (2.9)			1 (0.4)
GI.P2		1 (5.3)				1 (0.4)
GI.P3	2 (2.4)					2 (0.9)
GI.P7	2 (2.4)					2 (0.9)
GI.P11(Pb)	4 (4.9)					4 (1.8)
GII.P2	4 (4.9)					4 (1.8)
GII.P4	13 (15.9)	1 (5.3)	3 (8.8)			17 (7.6)
GII.P4 New Orleans	3 (3.7)	2 (10.5)	3 (8.8)			8 (3.6)
GII.P7			1 (2.9)			1 (0.4)
GII.P17	9 (11.0)	3 (15.8)	4 (11.8)			16 (7.1)
GII.P21	2 (2.4)	1 (5.3)	2 (5.9)			5 (2.2)
GII.P30				1 (1.4)		1 (0.4)
GII.P31(Pe)	3 (3.7)		1 (2.9)			4 (1.8)
GII.P33(Pg)	1 (1.2)					1 (0.4)
ORF2^*c*^						
GI.1	2 (2.4)		2 (5.9)			4 (1.8)
GI.4			7 (20.6)	1 (1.4)		8 (3.6)
GI.7	2 (2.4)					2 (0.9)
GII.2	5 (6.1)					5 (2.2)
GII.4 Sydney	3 (3.7)		1 (2.9)			4 (1.8)
GII.6	3 (3.7)					3 (1.3)
GII.7	1 (1.2)					1 (0.4)
GII.17	5 (6.1)					5 (2.2)
GII.21	1 (1.2)					1 (0.4)

Total	82	19	34	69	20	224

a Annual distribution of genotypes according to polymerase (ORF1) and capsid (ORF2) genes.

b ORF2 not determined.

c ORF1 not determined.

### Temporal distribution of noroviruses.

**FIG 1:**
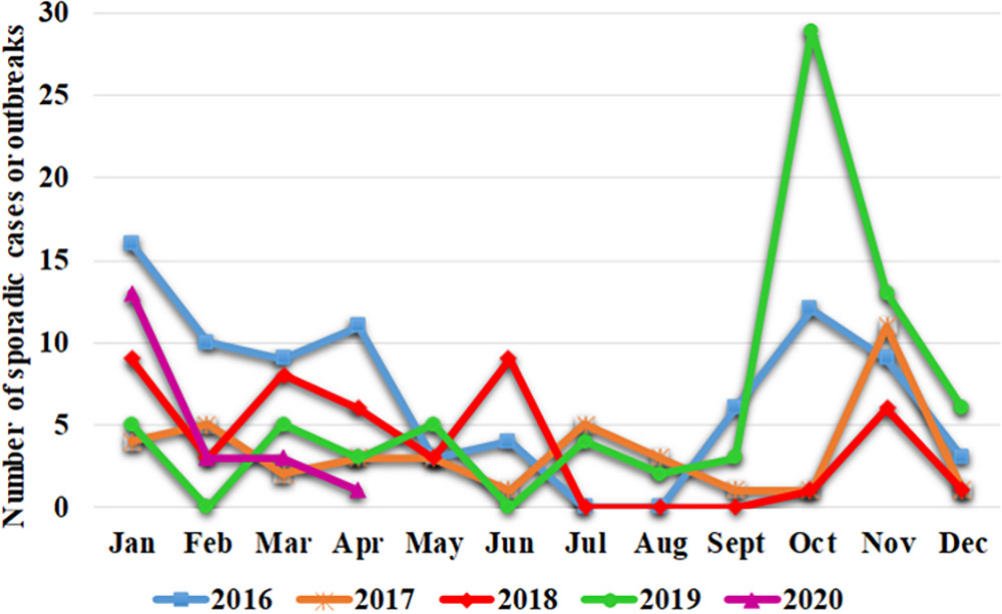
Distribution by month of norovirus infections detected from 2016 to 2020 in the Valencian Community, Spain.

Of the 264 norovirus cases, 83 (31.4%) were detected in 2016, 40 (15.2%) were detected in 2017, 46 (17.4%) were detected in 2018, 75 (28.4%) were detected in 2019, and 20 (7.6%) were detected between January and April 2020. The monthly distribution of acute gastroenteritis due to noroviruses from 2016 to 2020 showed clear seasonality (Fig. 1). The prevalence of norovirus infections was significantly higher during the first quarter of the year in 2016 and 2018 but during the last quarter in 2019 (*P < *0.05). Peaks of the epidemic curve were observed in January 2016 and 2018, in November 2017, and mainly in October 2019, while in contrast, few sporadic norovirus cases were registered during the summer months (July and August).

### Recombinant norovirus strains.

**FIG 2:**
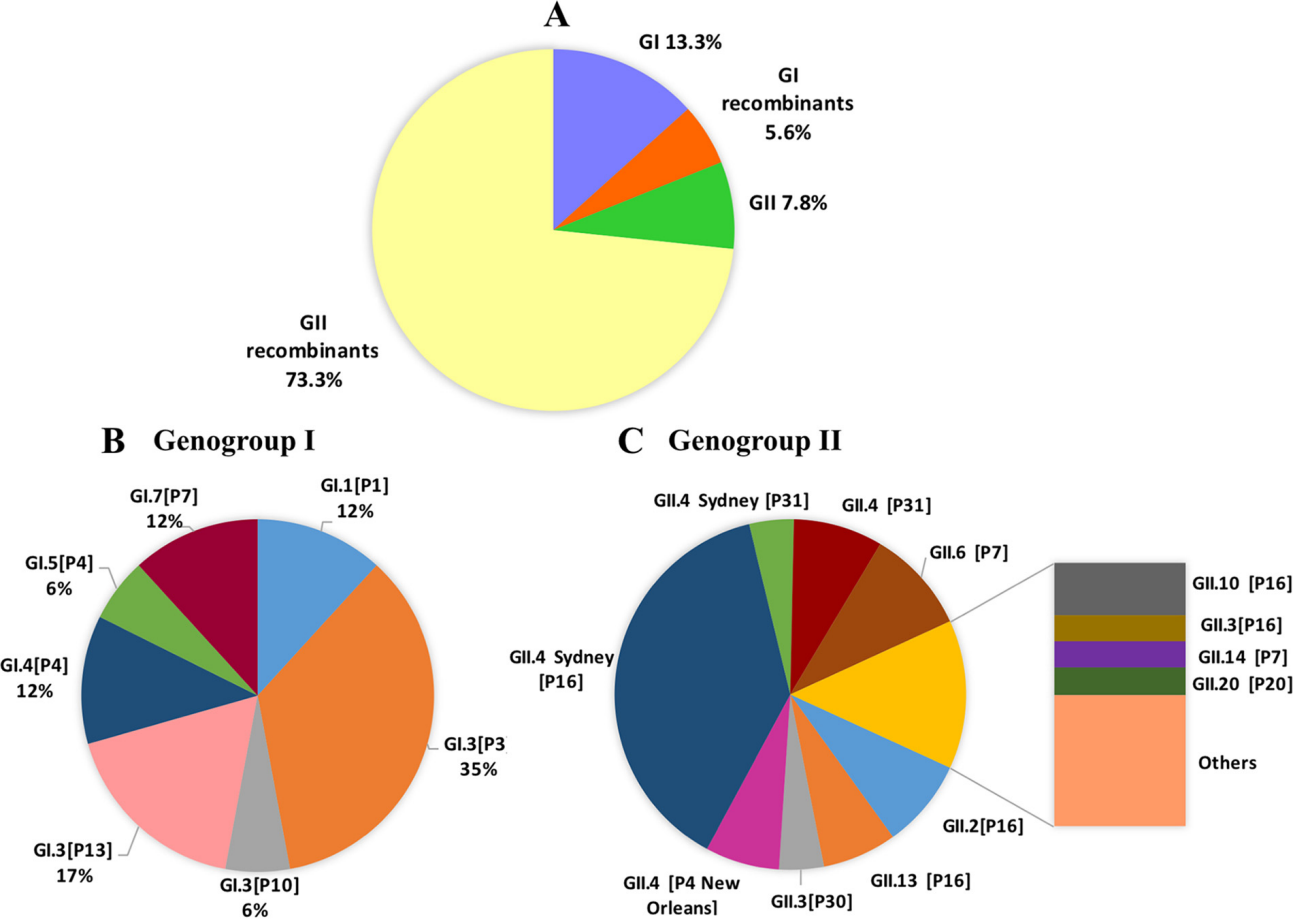
Diversity of norovirus genotypes from June 2018 to April 2020 in the Valencian Community, Spain. (A) Genogroup I, genogroup II, and recombinant strains. (B) Distribution of genogroup I strains. (C) Distribution of genogroup II strains. Others include GII.3[P12], GII.3[P21], GII.5[P40], GII.7[P7], and GII.12[P16].

From January 2016 to May 2018, six recombinant norovirus genotypes were detected, including GII.1[P33], GII.2[P16], GII.3[P16], GII.4 Sydney[P31], GII.6[P7], and GII.13[P21] (Table 1). From June 2018 onward, all norovirus samples positive by BD Max were routinely analyzed by sequencing of the ORF1/ORF2 junction to detect recombinant genotypes, yielding polymerase and capsid sequences for 90 norovirus strains detected between June 2018 and April 2020 (Fig. 2A). Recombinant strains within genogroup I were GI.3[P10], GI.3[P13], and GI.5[P4] (Fig. 2B). A remarkable number of recombinant strains belonged to genogroup II (90.4%; 66/73). Genotypes GII.4 Sydney[P16] (38.4%; 28/73), GII.6[P7] (7/73; 9.6%), GII.4[P31] (6/73; 8.2%), and GII.2[P16] (6/73; 8.2%) were found during the 2019–2020 season (Fig. 2C). Several capsid genotypes were associated with more than one polymerase type, including GI.3, GII.3, and GII.4 Sydney. Viruses harboring the GII.P16 polymerase were detected in 59% (43/73) of AGE cases. This GII.P16 polymerase was found among cases involving GII.2, GII.3, GII.4 Sydney 2012, GII.10, GII.12, and GII.13 viruses. Furthermore, noroviruses combined with the GII.P31(Pe) polymerase were associated with the GII.4 Sydney variant. Viruses with GII.P12, GII.P21, and GII.P30(Pc) polymerases were associated with GII.3.

### Genetic analysis of norovirus genogroup I.

**FIG 3:**
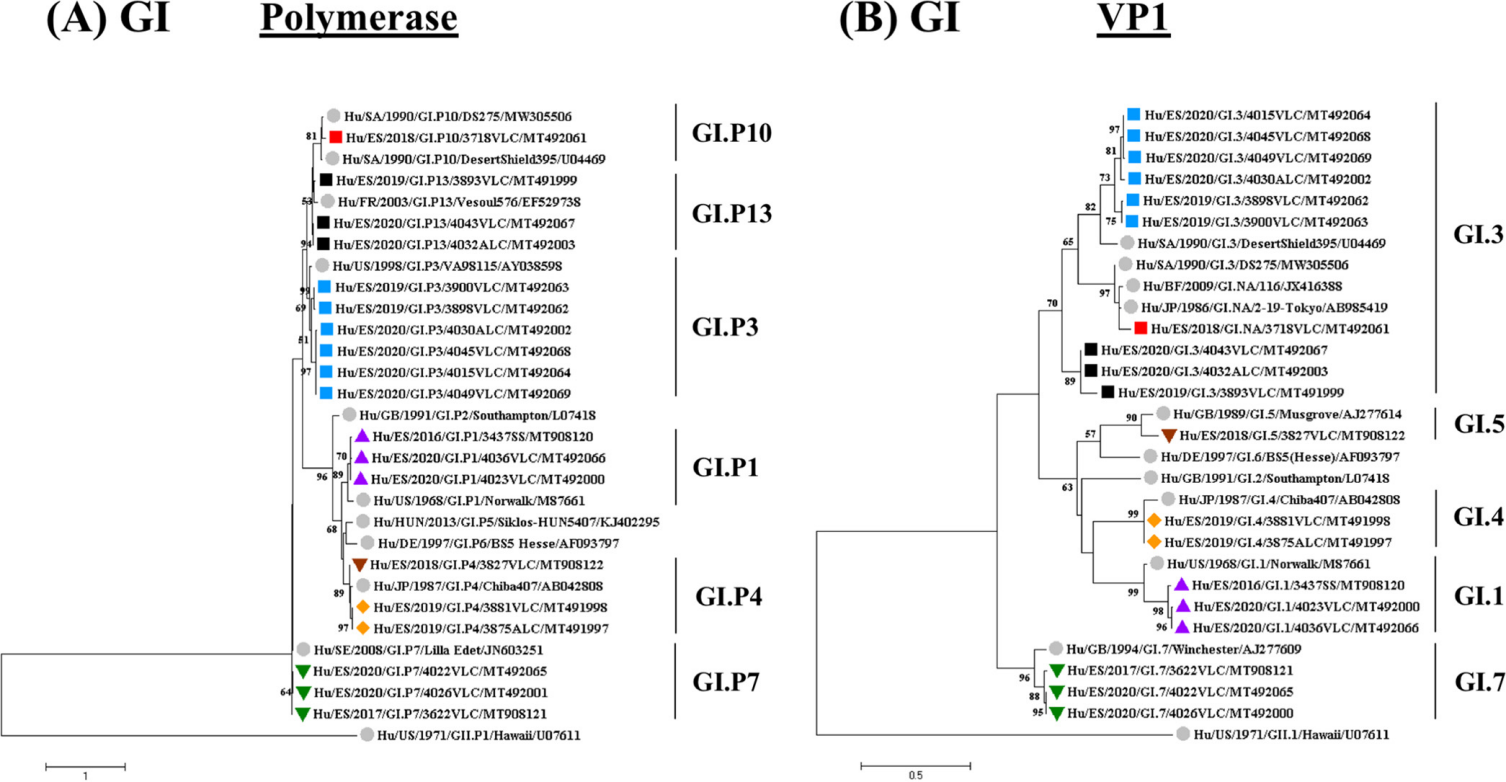
Phylogenetic analysis of GI norovirus based on partial polymerase and capsid genomic regions (VP1) from 2016 to 2020. (A) Phylogenetic tree of a 218-bp region of the polymerase gene including nucleotides 5157 to 5374 over the reference genome under GenBank accession number NC_044853. (B) Phylogenetic tree of a 244-bp region of VP1 including nucleotides 5374 to 5617 over the reference genome under GenBank accession number NC_044853. The trees were constructed using the maximum likelihood method based on the Kimura 2-parameter model with a bootstrap of 1,000 replicates. Bootstrap values of >50% are shown. The trees are drawn to scale; the branch lengths measure the number of substitutions per site. The analyses included 30 sequences (19 samples and 11 references). Reference strains are represented by their GenBank accession numbers and indicated with gray-filled circles. Norovirus strains reported in this study are indicated as follows: ▴ in purple, GI.1[P1]; ■ in blue, GI.3[P3]; ■ in red, GI.NA[P10]; ■ in black, GI.3[P13]; ♦ in orange, GI.4[P4]; ▾ in brown, GI.5[P4]; ▾ in green, GI.7[P7].

** (i) Phylogenetic analysis.** Phylogenetic analysis of GI norovirus strains based on partial polymerase and capsid region sequences from 2016 to 2020 was performed. The analysis included 30 sequences (19 samples and 11 references) (Fig. 3). Most of the strains detected were nonrecombinant (GI.1[P1], GI.3[P3], GI.4[P4], and GI.7[P7]), as shown in Fig. 3. Other recombinant genotypes such as GI.3[P10], GI.3[P13], and GI.5[P4] were also less frequently reported. It is noteworthy that isolate 3718 may represent a new capsid genotype. When genotyping was performed using the Norovirus Typing Tool (http://www.rivm.nl/mpf/norovirus/typingtool), no genotype was assigned to this capsid sequence. Furthermore, phylogenetic analysis of the partial capsid gene (Fig. 3B) showed that strain 3718 clusters with unassigned GI strains (GenBank accession numbers JX416388 and AB985419) reported by other authors (27, 28) and shares an ancestor with strain DS275 (accession number MW305506). Surprisingly, the recombinant GI.NA[P10] 3718 strain does not cluster with the reference Desert Shield strain (GenBank accession number U04469).

**FIG 4:**
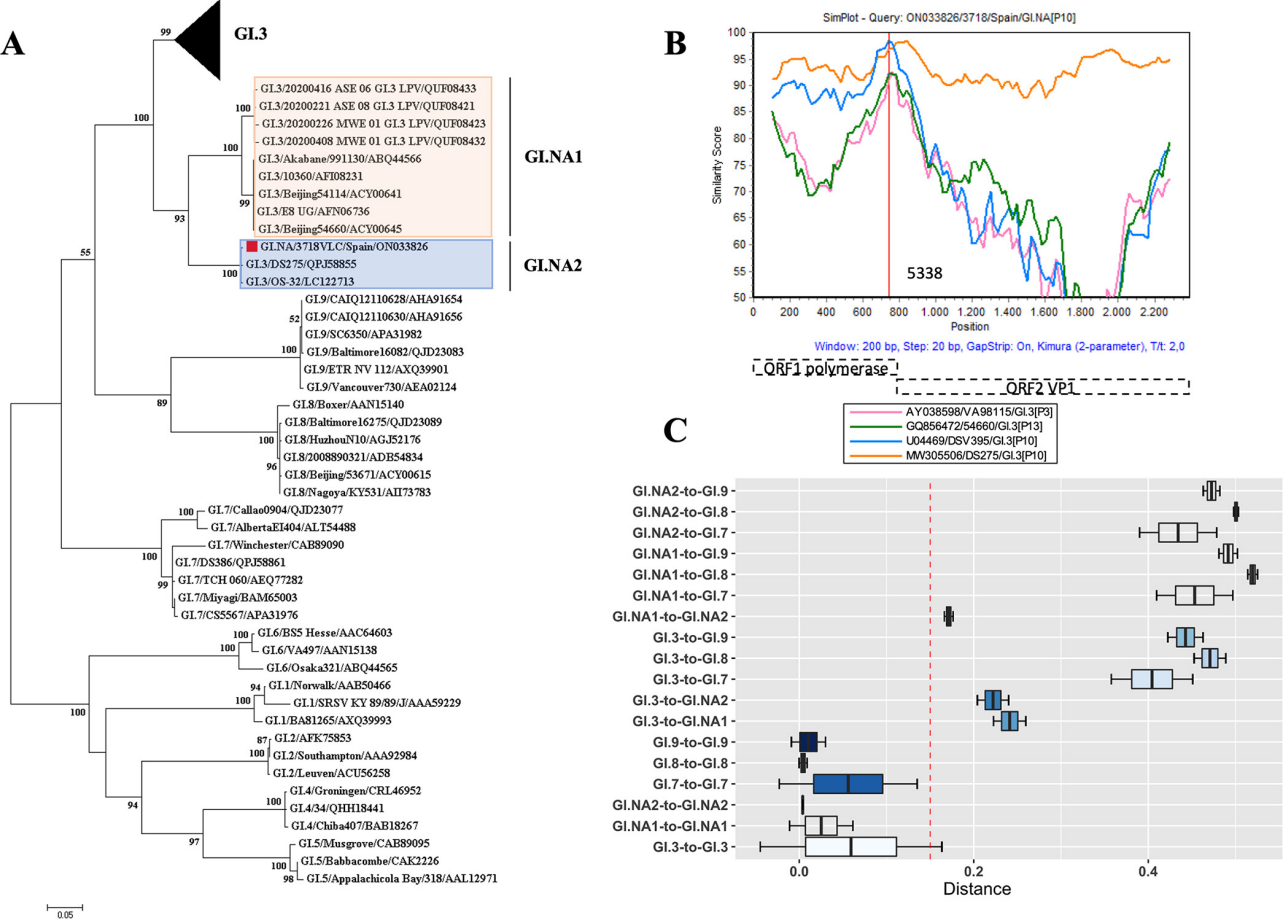
Phylogenetic analysis based on the full VP1 gene sequence of the GI.NA Spanish strain. (A) Phylogenetic tree of the complete major capsid protein (VP1). Reference strains are named after their protein identifier. The sequence obtained in this study is indicated by ■ in red (GI.NA[P10]) (GenBank accession number ON033826). The bar at the bottom of the tree indicates distance. The tree was constructed using the maximum likelihood method based on the Jones-Taylor-Thornton (JTT) matrix-based model with a bootstrap of 1,000 replicates. Bootstrap values of >50% are shown. (B) SimPlot analysis of Spanish GI.NA[P10] norovirus. A SimPlot was constructed using SimPlot version 3.5.1 with a sliding window width of 200 bp and a step size of 20 bp. The *x* axis indicates the nucleotide positions in the multiple alignments of the norovirus sequences; the *y* axis shows the percent similarity between norovirus reference strains and the query sequence. The recombination breakpoint is indicated in relation to the reference viral genome under GenBank accession number MW305506. (C) Patristic distance comparison of GI.NA1 and GI.NA2 viruses with their phylogenetically closer capsid genotypes. The *x* axis represents the comparisons of distances within and between capsid types. The black bar in the center of each box represents the average of the patristic distances, the edges of the box display 1×_SD_, and error bars show 2×_SD_. The red dashed line represents the 0.15 phylogenetic distance used as the cutoff threshold for new genotypes (for 2×_SD_ criteria, phylogenetic distances of sequences within a capsid type should not overlap distances between different capsid types).

**(ii) Proposal of two novel GI capsid genotype candidates.** The whole genome of isolate 3718 (GenBank accession number ON033826) was obtained by next-generation sequencing (NGS), and phylogenetic analysis of the complete VP1 amino acid sequence together with reference sequences was performed (Fig. 4A; see also Table S1 in the supplemental material). Three clearly defined clusters (GI.3, GI.NA1, and GI.NA2) were identified. Although these three clusters were grouped in a monophyletic branch, the analysis of patristic distances demonstrated that they are different genotypes (Fig. 4C). Criteria of 2 times the standard deviation (2×_SD_) were applied to classify GI.NA1 and GI.NA2 clusters (17). 2×_SD_ error bars of average distances within cluster GI.3 did not overlap 2×_SD_ of the mean distances between both GI.NA1 and GI.NA2 clusters compared to GI.3. Similarly, the distribution of patristic distance values for GI.NA1 and GI.NA2 did not overlap those of their closer genotypes GI.7, GI.8, and GI.9 (Fig. 4C). Accordingly, and using the same criteria, GI.NA1 and GI.NA2 represent two novel different genotypes. In addition, the mean phylogenetic-based distances of GI.NA1 (24.1%) and GI.NA2 (22.2%) with respect to the GI.3 group were above the 15% cutoff established by the Norovirus Classification Working Group (NCWG) to define new genotypes (14).

It is noteworthy that the two proposed genotypes include complete VP1 sequences from more than two geographic regions. GI.NA1 encompasses strains from China, South Korea, Japan, Vietnam, and Uganda, and GI.NA2 includes three sequences from Spain, Saudi Arabia, and Japan. Further investigation of possible changes within the two new proposed genotypes with regard to the GI.3 genotype was undertaken, and an alignment of the complete amino acid consensus sequences of VP1 was performed (Fig. S1). Many nonsynonymous amino acid changes were detected, most of them within a specific region of VP1 (amino acids [aa] 335 to 400), including major evolutionary changes such as insertions (threonine at aa 372) and deletions (aa 373) in the GI.NA1 and GI.NA2 genotypes, respectively. The two newly proposed genotypes (GI.NA1 and GI.NA2) showed two common deletions at amino acid positions 398 and 399 of VP1. Furthermore, SimPlot analysis was performed using the recombinant GI.NA[P10] virus (3718) as a query sequence (Fig. 4B). The data showed that GI.3[P10] Desert Shield (GenBank accession number U04469) is one of the parental sequences providing the polymerase region, and the DS275 strain (accession number MW305506), which was defined as a GI.3[P10] strain (29), is highly similar to the query recombinant strain 3718. In addition, the recombination breakpoint was in the ORF1 region (nucleotide position 5338 in relation to the reference viral genome under GenBank accession number MW305506). However, we did not identify the parental sequence providing the capsid region, and it was estimated that the nucleotide similarity ranged between 50 and 80% along VP1. Hence, according to 2×_SD_ criteria, we propose that the sequences within the GI.NA1 and GI.NA2 (including our isolate 3718) clusters should be redefined as new genotypes within genogroup I, which should be assigned as GI.10 and GI.11, respectively.

### Genetic analysis of norovirus genogroup II.

**FIG 5:**
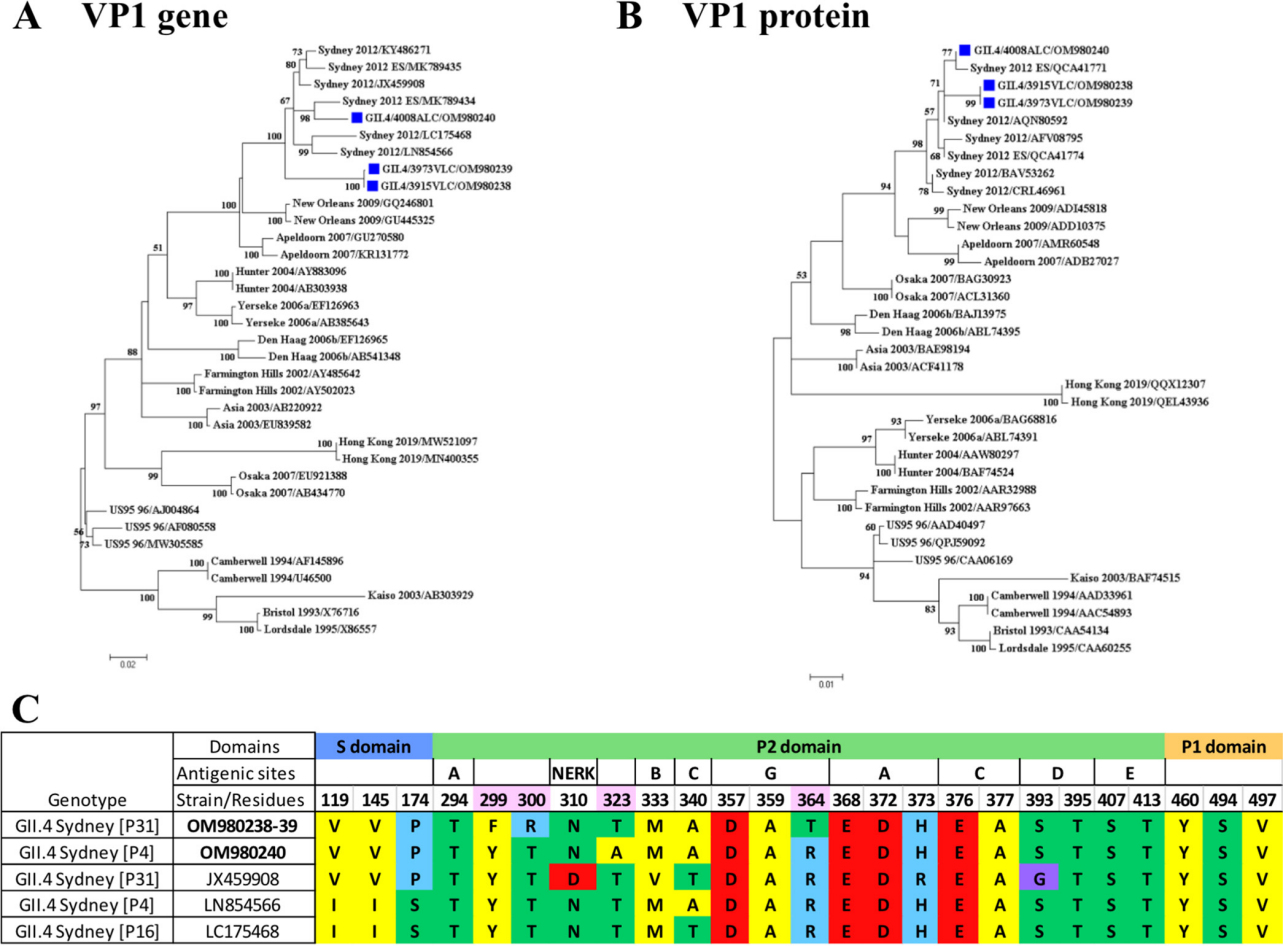
Characterization of norovirus GII.4 strains detected in this study. (A) Maximum likelihood tree of complete VP1 genes showing the evolution of all GII.4 variants. Spanish strains are identified in blue. (B) Same phylogenetic tree using the full VP1 protein. (C) Amino acid changes of GII.4 Sydney Spanish strains within the complete VP1 sequence (aa 1 to 540) compared to Sydney 2012 reference strains. Epitope binding regions A to E and the NERK motif are represented. Colors indicate the following amino acid categories: yellow, hydrophobic; green, uncharged; blue, positively charged; red, negatively charged; purple, special. Amino acid changes are in pink. GenBank accession numbers for Spanish strains are OM980238, OM980239, and OM980240.

**(i) Characterization of recombinant GII.4 viruses detected in this study.** Since 2016, three recombinant strains of the GII.4 Sydney variant have been identified as causative agents of gastroenteritis in our geographical area. GII.4 Sydney[P31] and GII.4 Sydney[P4 New Orleans] strains were predominant from 2016 to 2018, but in 2019, they were displaced by the recombinant GII.4 Sydney[P16] strain. Some GII.4 viral strains were found to be associated with P31 polymerase and were identified as unassigned variants by the Norovirus Typing Tool (version 2.0). NGS was performed to obtain the full genomes of two GII.4[P31] isolates (GenBank accession numbers OM980238 and OM980239) and one GII.4[P4 New Orleans] isolate (accession number OM980240). We analyzed the genetic variability of the GII.4 strains by performing phylogenetic analysis of full nucleotide (Fig. 5A) and amino acid (Fig. 5B) VP1 sequences, including all pandemic variants described for GII.4 (30). Regarding the VP1 gene phylogenetic tree, our GII.4 strains combined with P31 polymerase grouped separately within the GII.4 Sydney cluster. In contrast, these genetic differences were not found when the analysis was performed with the complete VP1 protein. As a result, our GII.4 strains have high similarity to GII.4 Sydney reference strains, grouping all strains in the same cluster (Fig. 5B). Therefore, these strains were eventually assigned to GII.4 Sydney. Four amino acid substitutions not previously reported were detected in our GII.4 strains within the P2 domain in the VP1 capsid protein (Fig. 5C). In GII.4[P31] strains, two amino acid changes (Y299F and T300R) occurred close to epitope A (aa 294 to 298). Another change was found at position 364 within epitope G (R364T). In GII.4[P4 New Orleans] strains, only one change was observed (T323A). The rest of the antigenic sites in VP1 were conserved.

**FIG 6:**
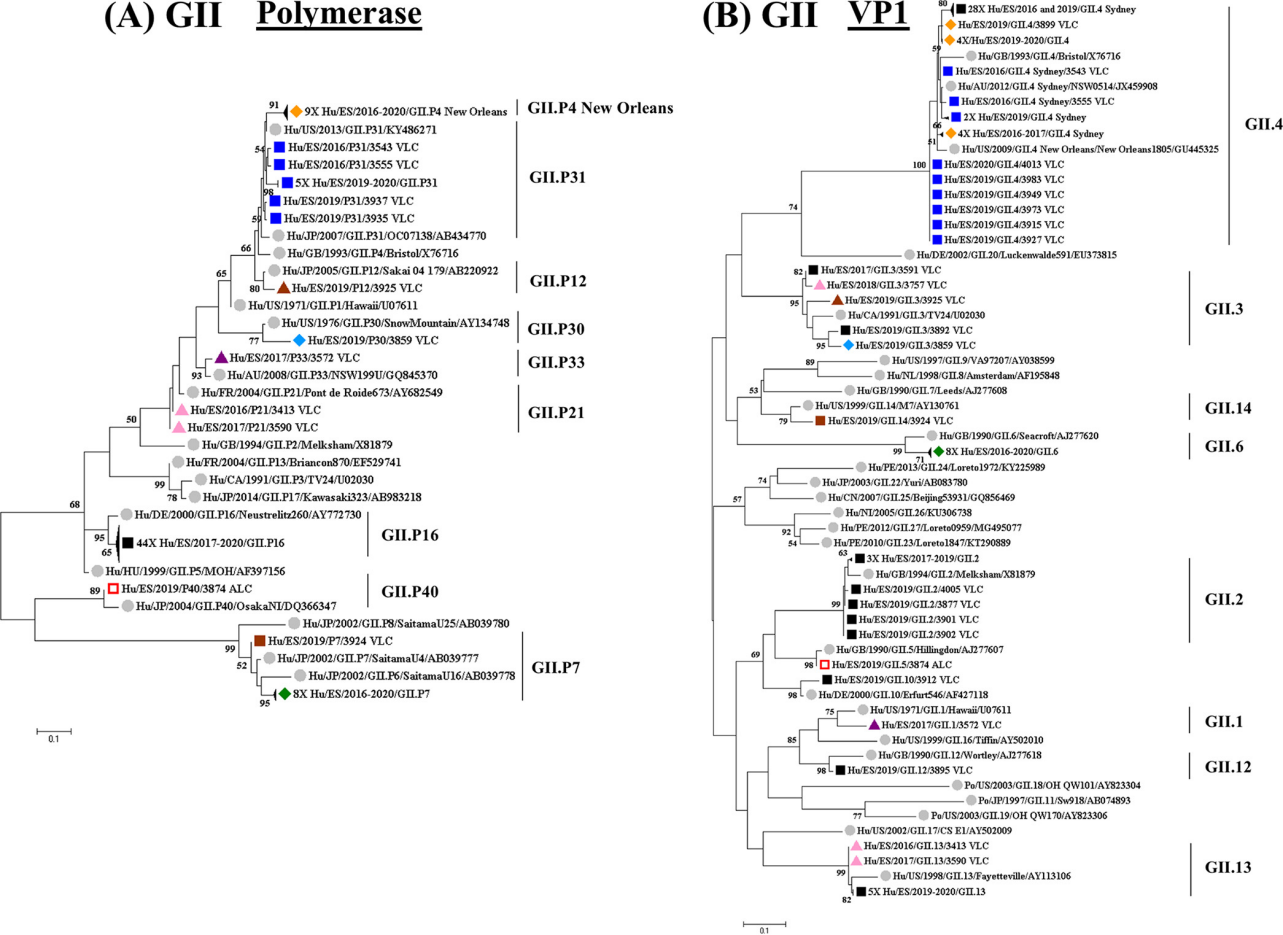
Phylogenetic analysis of human recombinant GII noroviruses based on partial polymerase (A) and VP1 (B) genomic regions. (A) Phylogenetic tree of a 209-bp region of polymerase including nucleotides 4876 to 5084 over the reference genome under GenBank accession number NC_029646. (B) Phylogenetic tree of a 290-bp region of VP1 including nucleotides 5085 to 5374 over the reference genome under GenBank accession number NC_039476. The trees were constructed using the maximum likelihood method based on the Kimura 2-parameter model with a bootstrap of 1,000 replicates. Bootstrap values of >50% are shown. The trees are drawn to scale; the branch lengths measure the number of substitutions per site. Reference strains are represented by their GenBank accession numbers and indicated with gray-filled circles. Norovirus strains reported in this study are indicated with the same colors in both phylogenetic trees.

**(ii) Molecular phylogenetic characteristics of recombinant GII noroviruses.** Phylogenetic analysis of partial polymerase (Fig. 6A) and VP1 (Fig. 6B) sequences confirmed the recombinant strains characterized in this study, corresponding to 15 different recombinant genotypes.

## DISCUSSION

Recombination is among the most important genetic-diversity-generating events within the *Norovirus* genus (19). The current study provides an overview of the molecular epidemiology of noroviruses drawn from an analysis of clinical samples collected in the Valencian Community, Spain, from January 2016 to April 2020. GII was the predominant genogroup (78.1%), with GII.4 Sydney 2012 being found to be the most prevalent genotype, followed by GII.2, GII.3, GII.6, and GII.17, which concurs with globally circulating strains (31, 32).

Here, we report norovirus infections caused by 18 different recombinant strains for the first time in Spain, including uncommon genotypes such as GI.5[P4] and GII.5[P40]. A high prevalence of recombinant strains (90.4%) was observed among genogroup II viruses from 2018 to 2020. In contrast, only five recombinant strains were detected in a previous study carried out from 2009 to 2012 in the Basque Country, Spain (33). Remarkably, most outbreaks during the 2019–2020 period were caused by GII.4 Sydney strains associated with P16 and P31 polymerases. The emergence of these strains coincided with a high prevalence of GII.2[P16] viruses in different Asian and European countries (34, 35). Note that the emergence of these genotypes did not lead to major amino acid changes in the capsid of GII.4 (36, 37), suggesting that the evolutionary advantage lies in the polymerase. Interestingly, in this study, we found GII.P16 polymerase harboring six different capsids (GII.2, GII.3, GII.4 Sydney, GII.10, GII.12, and GII.13). However, this “promiscuity” was not unique to the P16 polymerase, as P21 was also detected in combination with GII.3 and GII.13. We also found GI.3 and GII.3 viruses associated with different polymerases (P10 and P13 and P12, P21, and P30, respectively). The versatility of multiple polymerases in combination with different capsids supports the idea that the polymerase plays a crucial role in norovirus infection. Furthermore, a recent evolutionary analysis of 25 different polymerases pinpointed high evolutionary rates in P4, P12, P16, and P31 strains, suggesting that the polymerase region evolves rapidly, similarly to VP1 (38). A meta-analysis of norovirus infections recently carried out in China showed that recombinant strains carrying the GII.P16 polymerase genotype, such as GII.2 and GII.4, have caused epidemics, and those authors concluded that the GII.P16 polymerase may trigger pandemics (39).

In the present study, we identified several GII.4 Sydney strains associated with the P31 polymerase, which cluster together in the phylogenetic tree of the complete VP1 gene of GII.4 (Fig. 5A). Further analyses of ORF2 of these isolates were performed to examine possible amino acid changes in antigenic epitopes of VP1 (Fig. 5C). As a result, three novel amino acid changes were described (Y299F, T300R, and R364T), and two of them occurred near epitope A (aa 294 to 298) (40). Epitope A is an important vaccine target considered an evolving GII.4 antibody blockade epitope and is located close to the histo-blood group antigen (HBGA) binding pocket (41). Changes close to epitope A could allow the virus to evade the immune response. A new GII.4 variant could emerge if strains from this subcluster continue to evolve and spread worldwide, accumulating capsid changes. A recent study has shown that pandemic GII.4 variants require preadaptation to the host, circulating in the population for up to 9 years before emerging globally (42). This further supports the importance of epidemiological surveillance of norovirus infections.

In genogroup I, GI.3 and GI.4 viruses were the most common genotypes in this study, in agreement with previous reports (43, 44). The presence of recombinant strains is lower in this genogroup than in GII, although we observed both GI.3[P13] and GI.3[P10] as well as the more uncommon GI.5[P4]. The widespread circulation of GI.3[P13] strains has been reported in Taiwan (45). Nevertheless, there is little awareness of emerging new variants among GI viruses. Here, we have identified an isolate (3718 [GenBank accession number ON033826]) that could represent a new capsid genotype. Phylogenetic analysis of the complete VP1 amino acid sequence of isolate 3718 (GenBank accession number ON033826) together with 66 GI.3 reference sequences showed three separate clusters: GI.3, GI.NA1, and GI.NA2 (Fig. 4A). Patristic analysis (Fig. 4C) demonstrated that based on the VP1 amino acid sequence, the number of genotypes within genogroup I should be expanded with two new genotypes. As evidenced previously, we propose that the GI.NA1 and GI.NA2 clusters should be considered novel genotype candidates within genogroup I.

It is worth noting that although GI.3 is a static capsid genotype (26), it was recently shown to have a large number of sites under positive pressure, which could explain the emergence of new genotypes (29). In addition, GI norovirus surveillance should be encouraged by establishing classification criteria for new variants within genotypes.

In summary, this study reports a diverse range of recombinant norovirus strains circulating in Spain from 2016 to 2020 and describes two tentative new capsid GI genotypes. A total of 18 recombinant strains have been detected, an increase that may be due to improved genotyping procedures such as dual-typing assays. These changes have highlighted the need to unify detection criteria for noroviruses targeting the ORF1/ORF2 overlapping region in order to characterize the genetic diversity of recombinant noroviruses worldwide.

## MATERIALS AND METHODS

### Sample collection.

The presence of noroviruses was investigated in specimens from patients with sporadic gastroenteritis attending primary care clinics and the emergency department of the Hospital Clínico Universitario of Valencia. A total of 4,950 stool samples were tested by conventional RT-PCR and/or RT-qPCR between January 2016 and April 2020. The study was approved by the Clinical Research Ethics Committee of the Hospital Clínico Universitario of Valencia. Additionally, samples from norovirus gastroenteritis outbreaks occurring at different Spanish locations (Alicante, Castellón, Cádiz, San Sebastián, and Palma de Mallorca) were analyzed anonymously. However, the total number of samples from other geographical areas was low (2.9%). All specimens were stored at 4°C until further molecular analysis.

### RNA extraction and reverse transcription.

Viral RNA was extracted from 10% fecal suspensions in phosphate-buffered saline (PBS) with TRIzol reagent (Invitrogen) (46). The reverse transcription reaction was performed with random primers using SuperScript III reverse transcriptase (Invitrogen).

### Norovirus detection.

From January 2016 to May 2018, viral detection was carried out by conventional PCR, amplifying the polymerase gene (primer pair JV12/JV13) and/or the capsid gene (primer pairs G1SKF/G1SKR and G2SKF/G2SKR for GI and GII, respectively) (47, 48). Amplicons were analyzed by electrophoresis on 1.5% agarose gels with RedSafe staining (RedSafe nucleic acid staining solution; iNtRON Biotechnology). After June 2018, the presence of noroviruses and other enteric viruses in stool samples was determined using the BD Max enteric viral panel on the BD Max system (Becton, Dickinson).

### Detection and analysis of recombinant strains.

The overlapping ORF1/ORF2 junction region was sequenced to identify recombinant norovirus strains. In brief, RT-PCR was performed with a combination of the following previously described oligonucleotides: primers MON 432 (TGG ACI CGY GGI CCY AAY CA) and G1SKR (CCA ACC CAR CCA TTR TAC A) for genogroup I and primers MON 431 (TGG ACI AGR GGI CCY AAY CA) and G2SKR (CCR CCN GCA TRH CCR TTR TAC AT) for genogroup II (48, 49). RT-PCR was performed using Qiagen one-step RT-PCR kit master mix, with 20 U of an RNase inhibitor (Biotools) and the following thermal cycling conditions: 30 min at 50°C, 15 min at 95°C, and 40 cycles of 95°C for 30 s, 50°C for 30 s, and 72°C for 45 s, followed by 7 min at 72°C.

### Sequencing and genotyping methods.

The PCR products were purified by using an NZYGelpure kit (Nzytech) and sequenced upstream and downstream. Sequence quality was checked with BioEdit software v7.0.0 (50). The viral genogroup, genotype, and variant were assigned by using the Norovirus Typing Tool (http://www.rivm.nl/mpf/norovirus/typingtool) and the Human Calicivirus Typing Tool (http://norovirus.ng.philab.cdc.gov/). The strains were named according to the newly proposed dual-typing designations indicating the genotype of the capsid followed by the polymerase type in brackets (17).

### Next-generation sequencing.

The complete genomic viral RNA of selected norovirus strains was obtained by next-generation sequencing (NGS) analysis at the Central Service for Experimental Research of the University of Valencia. Libraries were constructed using the TruSeq mRNA library prep kit (Illumina) and sequencing on a MiSeq platform (Illumina). Raw-read quality control was assessed with FastQC version 0.11.5 (51). Reads were quality trimmed using seqtk trimfq version 1.2-r101-dirty (52), and genomes were assembled as previously described (53–55).

### Data and phylogenetic analyses.

The temporal distributions of norovirus infections per quarter and year were statistically analyzed with GraphPad Prism software version 9.3.0. Pearson’s chi-squared test was performed to evaluate monthly quarters’ sporadic cases for which the proportion for each year differed from a hypothetical proportion of 25%.

Phylogenetic trees were built with the sequences from this study and reference sequences obtained from GenBank. Sequences were aligned with Clustal X 2.0 (56) and equated with GeneDoc 2.7.000 (57). Finally, phylogenetic analyses were performed using MEGA7 (Molecular Evolutionary Genetics Analysis v7.0) (58). The MEGA maximum likelihood model selection tool and Bayesian information criterion were used to determine the best nucleotide/amino acid substitution model. Furthermore, the best model suggested by the program was used to calculate the level of nucleotide sequence identity between the sequences studied. The evolutionary history was inferred by the maximum likelihood method (59) using a bootstrap test with 1,000 replicates to evaluate tree reliability. New tentative genotypes were determined based on a difference in the VP1 amino acid composition above 15% and the classification criterion applying the standard deviation (SD) of average patristic distances within and between phylogenetic clusters based on VP1 ML trees (14, 17) using R (60) and the adephylo package (61). Patristic distances were plotted by employing the R package ggplot2 (62). All the code and data sets used to calculate patristic distances and generate the plot are available in the GitHub repository (https://github.com/gastrovirus-uv/navarro-lleo_etal_norovirusGI_genotypes).

### Data availability.

Norovirus sequences derived in this study were deposited in GenBank under accession numbers MK789431, MK789432, MN918435 to MN918437, MN854082 to MN854085, MT491997 to MT492003, MT492038 to MT492049, MT492061 to MT492069, MT495616 to MT495630, MT495732 to MT495748, MT501813 to MT501864, MT908120 to MT908122, MT908849 to MT908867, ON418988 to ON418998, ON506826 to ON506874, ON506878 to ON506882, ON506919, ON506929 to ON506936, ON507067 to ON507069, and ON507283 to ON507286. Complete consensus genome sequences and raw NGS reads obtained in this study were deposited in GenBank (accession numbers OM980238 to OM980240 and ON033826) and the Sequence Read Archive (SRA) (BioProject accession number PRJNA814216), respectively.
