# Supplementary material for: Recombinant Noroviruses Circulating in Spain from 2016 to 2020 and Proposal of Two Novel Genotypes within Genogroup I
Source: Microbiol Spectr. 2022 Jul 13;10(4):e02505-21. doi: 10.1128/spectrum.02505-21 (PMC9430863; doi:10.1128/spectrum.02505-21)
Supplement: Supplemental file 1 — Table S1 and Fig. S1. Download spectrum.02505-21-s0001.pdf, PDF file, 0.2 MB [file spectrum.02505-21-s0001.pdf]

Table S1: Norovirus VP1 sequences downloaded from GenBank for Figure 4 in this study.

Accesion number	Protein id	Strain	Genotype	Country	Year
M87661	AAB50466	Norwalk	GI.1	United States	1968
L23828	AAA59229	SRSV-KY-89	GI.1	Japan	1989
MH638229	AXQ39993	BA81265	GI.1	United States	2017
L07418	AAA92984	Southampton	GI.2	United Kingdom	1991
FJ515294	ACU56258	Leuven	GI.2	Belgium	2003
JQ743332	AFK75853	GI.2	GI.2	United States	1999
MW445537	QQL02664	18N239	GI.3	China	2018
MW600284	QRQ46993	CFIA-FV-478-13_43479-9	GI.3	Canada	2017
MW600279	QRQ46942	CFIA-FV-448-1b_ 43479-8	GI.3	Canada	2017
MW590348	QRM14432	CFIA-FV-491-23_43538-14	GI.3	Canada	2017
MW590347	QRM14429	CFIA-FV-491-16_43538-5	GI.3	Canada	2017
MW590338	QRM14330	CFIA-FV-340-1.3_43479-1234	GI.3	Canada	2017
MW558948	QRD99198	CFIA-FVR-002	GI.3	Canada	2012
MZ462932	QXD98784	G7_8	GI.3	Mexico	2017
MZ462931	QXD98781	G5_10	GI.3	Mexico	2016
MZ462930	QXD98778	G5_9	GI.3	Mexico	2016
MZ462929	QXD98775	G2_12	GI.3	Mexico	2016
MZ021661	QUF08433	20200416_ASE_06_GI.3_LPV	GI.3	South Korea	2020
MZ021660	QUF08432	20200408_MWE_01_GI.3_LPV	GI.3	South Korea	2020
MZ021651	QUF08423	20200226_MWE_01_GI.3_LPV	GI.3	South Korea	2020
MZ021649	QUF08421	20200221_ASE_08_GI.3_LPV	GI.3	South Korea	2020
MK073893	AZJ17766	Nashville-0047	GI.3	United States	2016
MK073892	AZJ17763	Nashville-0046	GI.3	United States	2016
MK073887	AZJ17748	BayCity-0088	GI.3	United States	2016
KT781411	AOO95034	EN-3	GI.3	Taiwan	2015
U04469	AAA16285	DSV395	GI.3	Saudi Arabia	1990
JN699050	QPJ58810	B8	GI.3	:ntral African Repub	1977
JN699049	AFN06738	C9	GI.3	French Guiana	1978
JN699048	AFN06737	C91	GI.3	French Guiana	1978
JN699047	AFN06736	E8_UG	GI.3	Uganda	1976
JQ911594	AFI08231	10360	GI.3	Viet Nam	2010
GQ856473	ACY00647	Beijing55042	GI.3	China	2007
GQ856472	ACY00645	Beijing54660	GI.3	China	2007
GQ856471	ACY00641	Beijing54114	GI.3	China	2007
EF547396	ABQ44566	Akabane/991130	GI.3	Japan	1999
LC378993	BBD74626	FS118-2015	GI.3	Japan	2015
LC378992	BBD74624	FS114-2015	GI.3	Japan	2015
LC378991	BBD74622	FE61-2015	GI.3	Japan	2015
LC378990	BBD74620	FE59-2015	GI.3	Japan	2015
LC378989	BBD74618	FE47-2015	GI.3	Japan	2015
LC378988	BBD74616	FE29-2015	GI.3	Japan	2015
LC378987	BBD74614	FE8-2015	GI.3	Japan	2015
LC378986	BBD74612	FE2-2015	GI.3	Japan	2015
LC378985	BBD74610	FS421-2014	GI.3	Japan	2015
LC378984	BBD74608	FE49-2014	GI.3	Japan	2015
MH218729	AWR17846	NORO_90_22_06_2015	GI.3	United Kingdom	2015
MH218728	AWR17843	NORO_89_01_04_2015	GI.3	United Kingdom	2015

Accesion number	Protein id	Strain	Genotype	Country	Year
MH218727	AWR17840	NORO_87_18_02_2015	GI.3	United Kingdom	2015
MH218726	AWR17837	NORO_85_14_11_2014	GI.3	United Kingdom	2014
MH218725	AWR17834	NORO_84_04_11_2014	GI.3	United Kingdom	2014
MH218724	AWR17831	NORO_83_31_10_2014	GI.3	United Kingdom	2014
MH218722	AWR17825	NORO_81_10_10_2014	GI.3	United Kingdom	2014
MH218664	AWR17651	NORO_206_03_12_2015	GI.3	United Kingdom	2015
MH218659	AWR17636	NORO_198_10_11_2015	GI.3	United Kingdom	2015
MH218647	AWR17600	NORO_179_03_08_2015	GI.3	United Kingdom	2015
KY934262	ARI71147	0304-19	GI.3	China	2015
AF414405	AAL12968	Little_Rock/316	GI.3	United States	1994
AF414403	AAL12962	Honolulu/219	GI.3	United States	1992
AY038598	AAK84673	VA98115	GI.3	United States	1998
KJ196292	AII73765	Shimizu/KK2866	GI.3	Japan	2007
KJ194510	AHV83751	Amsterdam_2_1995	GI.3	Netherlands	1995
JQ743330	AFK75851	GI.3	GI.3	United States	1999
AF439267	AAL32455	Potsdam_196	GI.3	Germany	2000
EF547393	ABQ44563	Osaka/010314	GI.3	Japan	2001
AF145709	AAD37377	Stav/95	GI.3	Norway	1995
FJ711164	ACX33983	JKPG_883	GI.3	Sweden	2007
FJ711163	ACX33982	JKPG_881	GI.3	Sweden	2007
AJ277612	CAB89093	Birmingham	GI.3	United Kingdom	1993
MT031988	QHW16218	CS0048	GI.3	United States	2019
MN448477	QEP29903	Westport0085	GI.3	United States	2013
LC122713	-	OS-32	GI.3	Japan	2012
MW305506	QPJ58855	DS275	GI.3	Saudi Arabia	1990
AB042808	BAB18267	Chiba407	GI.4	Japan	1987
LN854563	CRL46952	Groningen	GI.4	Netherlands	2011
MN938461	QHH18441	34	GI.4	China	2019
AJ277614	CAB89095	Musgrove	GI.5	United Kingdom	1989
AF414406	AAL12971	Appalachicola_Bay/318	GI.5	United States	1995
AM263418	CAK2226	Babbacombe	GI.5	United Kingdom	1996
AF093797	AAC64603	BS5_Hesse	GI.6	Germany	1997
AF538678	AAN15138	VA497	GI.6	United States	1999
EF547395	ABQ44565	Osaka321	GI.6	Japan	2000
AJ277609	CAB89090	Winchester	GI.7	United Kingdom	1994
MT372469	QJD23077	Callao0904	GI.7	United States	2017
MW305508	QPJ58861	DS386	GI.7	Saudi Arabia	1990
JN005886	AEQ77282	TCH-060	GI.7	United States	2003
AB758449	BAM65003	Miyagi	GI.7	Japan	2009
KX907729	APA31976	CS5567	GI.7	United States	2011
KU311161	ALT54488	AlbertaEI404	GI.7	Canada	2012
AF538679	AAN15140	Boxer	GI.8	United States	2001
GU299761	ADB54834	2008890321	GI.8	United States	2008
KC473548	AGJ52176	HuzhouN10	GI.8	China	2008
GQ856462	ACY00615	Beijing53671	GI.8	China	2007
KJ196298	AII73783	NagoyaKY531	GI.8	Japan	2007
MT372476	QJD23089	Baltimore16275	GI.8	United States	2010
HQ637267	AEA02124	Vancouver730	GI.9	Canada	2004
KF586507	AHA91654	CAIQ12110628	GI.9	China	2012

Accesion number	Protein id	Strain	Genotype	Country	Year
KF586508	AHA91656	CAIQ12110630	GI.9	China	2012
KX907731	APA31982	SC6350	GI.9	United States	2016
MH702262	AXQ39901	ETR-NV-112	GI.9	Nepal	2014
MT372474	QJD23083	Baltimore16082	GI.9	United States	2013

```
                              10        20        30        40        50        60        70        80        90       100
                    ....|....|....|....|....|....|....|....|....|....|....|....|....|....|....|....|....|....|....|....|
GI.3_consensus      MMMASKDAPTNMDGTSGAGQLVPEANTAEPISMEPVAGAATAAATAGQVNMIDPWIMNNYVQAPQGEFTISPNNTPGDILFDLQLGPHLNPFLSHLAQMY
GI.NA1_consensus    .................................D........V.....I........S.F.........V........V........Q.....A......
GI.NA2_consensus    ...............................A.D..V.....V..............S.F...........................Q............

                             110       120       130       140       150       160       170       180       190       200
                    ....|....|....|....|....|....|....|....|....|....|....|....|....|....|....|....|....|....|....|....|
GI.3_consensus      NGWVGNMKVKVLLAGNAFTAGKIIISCIPPGFAAQNISIAQATMFPHVIADVRVLEPIEVPLEDVRNVLFHNNDNTPTMRLVCMLYTPLRASGSSSGTDP
GI.NA1_consensus    .......R........................TS.......M.................I...........T...R...............N........
GI.NA2_consensus    .......R..I.....................TS.SV....M.....I...........I...........T...R...............N........

                             210       220       230       240       250       260       270       280       290       300
                    ....|....|....|....|....|....|....|....|....|....|....|....|....|....|....|....|....|....|....|....|
GI.3_consensus      FVIAGRVLTCPSPDFSFLFLVPPNVEQKTKPFSVPNLPLNTLSNSRVPSLIKSMMVSRDHGQMVQFQNGRVTLDGQLQGTTPTSASQLCKIRGSVFHANG
GI.NA1_consensus    ...........DSN...............R......I.................TI...QN.II....................V........TTY..T.
GI.NA2_consensus    ...........DST.N..............Q.........S..............I...QA.VI........................F.L..T..T.S.

                             310       320       330       340       350       360       370       380       390       400
                    ....|....|....|....|....|....|....|....|....|....|....|....|....|....|....|....|....|....|....|....|
GI.3_consensus      GNGYNLTELDGSPYHAFESPAPIGFPDLGECDWHMEASPTTQFNTGDVIKQINVKQESAFAPHLGTVQVDG-LSDVSVNTNMIAKLGWVSPVSDGHKGDV
GI.NA1_consensus    .S.I.....N.E.................G....LT.T..QA..D.AKVVRLS.T.GA........IHYTTTDT.YNP..SI.CT.D.L.QTAGQNN--.
GI.NA2_consensus    .K.L.......T...............I.D....LI.V...T.PNDGHNRE.A.T.G........SIHFT.--G.LNN.D.V.CSIN.I..IDAVK.--.

                             410       420       430       440       450       460       470       480       490       500
                    ....|....|....|....|....|....|....|....|....|....|....|....|....|....|....|....|....|....|....|....|
GI.3_consensus      DPWVIPRYGSTLTEAAQLAPPIYPPGFGEAIVFFMSDFPIAHGTNGLSVPCTIPQEFVTHFVNEQAPTRGEAALLHYLDPDTNRNLGEFKLYPDGFMTCV
GI.NA1_consensus    ...Q..T...............F......TL...L.....SN.K........L..............I.........V....H..........E......
GI.NA2_consensus    N.....N......................T.......G...N.L........L........................I....H.......I..E......

                             510       520       530       540
                    ....|....|....|....|....|....|....|....|....|.
GI.3_consensus      PNSSGTGPQTLPINGVFVFVSWVSRFYQLKPVGTAGPARRLGIRRS
GI.NA1_consensus    ..T..G..............................A.........
GI.NA2_consensus    ..A..S...........T..................AV........
```

Supplementary Figure S1. Alignment of complete VP1 amino acid sequence of GI.3, GI.NA1 and GI.NA2 genotypes. Amino acids are shown according to the RasMol amino acid scheme colours. Dots represent conserved residues and black boxes show deletions and insertions with respect to the GI.3 consensus sequence.
